# Supplementary material for: Intravitreal injections of corticosteroid and the risk of central serous chorioretinopathy
Source: PLoS One. 2026 Mar 30;21(3):e0343704. doi: 10.1371/journal.pone.0343704 (PMC13035135; doi:10.1371/journal.pone.0343704)

**S1 Fig. Detailed optical coherence tomography and clinical findings of three suspected central serous chorioretinopathy cases identified in Clinical Data Warehouse screening.**

(A) Case 1: Posterior uveitis initially presenting with subretinal fluid that was suspected as CSC. Fundus examination revealed anterior chamber cells and sclerotic vascular changes. Fluorescein angiography demonstrated peripapillary vascular leakage without characteristic focal leak of CSC. Intravitreal triamcinolone acetonide injection was done. (B) Case 2: Pseudophakic cystoid macular edema with secondary subretinal fluid developing 5 months after uncomplicated cataract surgery. Initial treatment with intravitreal bevacizumab showed no improvement with increasing intraretinal fluid. Complete resolution of both subretinal and intraretinal fluid occurred after intravitreal triamcinolone acetonide injection, consistent with pseudophakic cystoid macular oedema rather than primary CSC. Top row shows baseline presentation, middle row shows post-bevacizumab worsening, and bottom row shows resolution after triamcinolone injection. CSC = central serous chorioretinopathy.


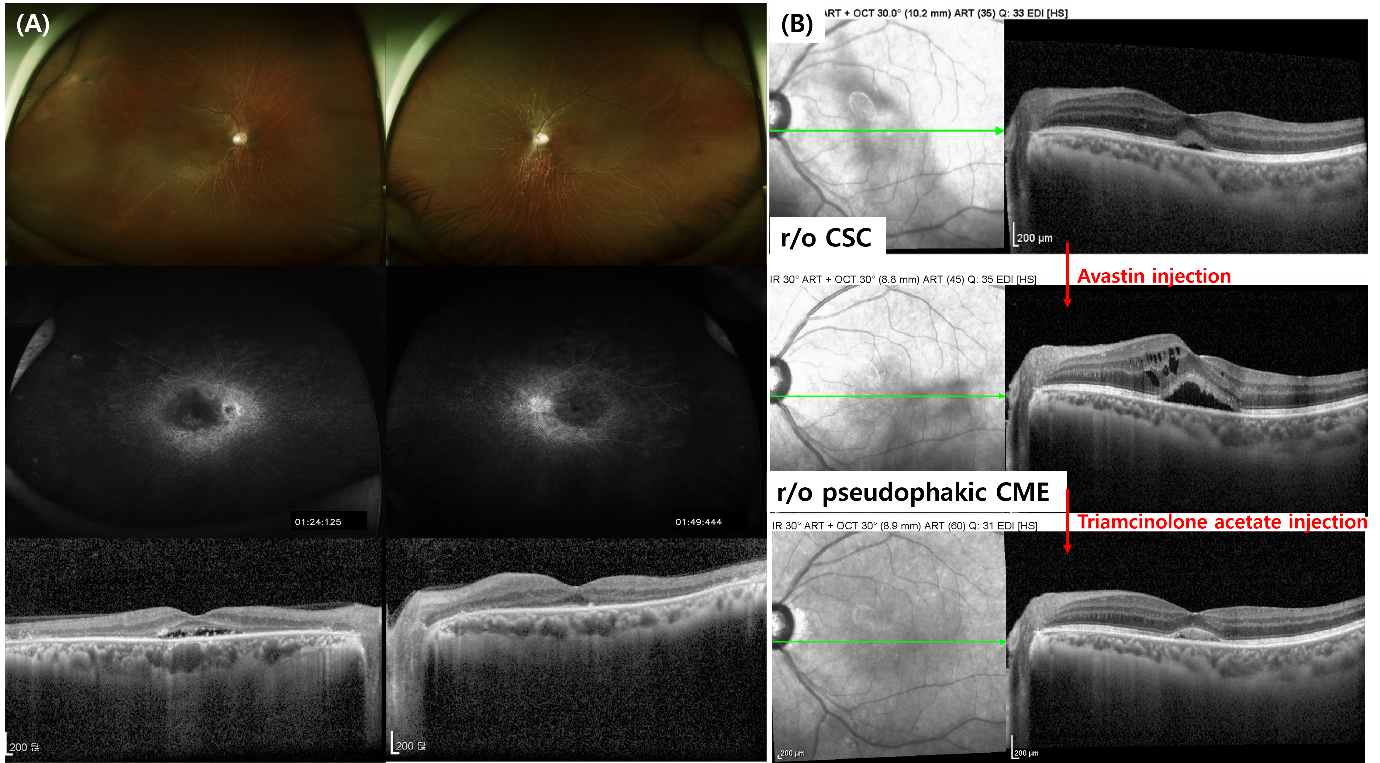

Supplement: S1 Fig — (A) Case 1: Posterior uveitis initially presenting with subretinal fluid that was suspected as CSC. Fundus examination revealed anterior chamber cells and sclerotic vascular changes. Fluorescein angiography demonstrated peripapillary vascular leakage without characteristic focal leak of CSC. Intravitreal triamcinolone acetonide injection was done. (B) Case 2: Pseudophakic cystoid macular edema with secondary subretinal fluid developing 5 months after uncomplicated cataract surgery. Initial treatment with intravitreal bevacizumab showed no improvement with increasing intraretinal fluid. Complete resolution of both subretinal and intraretinal fluid occurred after intravitreal triamcinolone acetonide injection, consistent with pseudophakic cystoid macular oedema rather than primary CSC. Top row shows baseline presentation, middle row shows post-bevacizumab worsening, and bottom row shows resolution after triamcinolone injection. CSC = central serous chorioretinopathy. (DOCX) [file pone.0343704.s001.docx]
