# Supplementary material for: Intravitreal injections of corticosteroid and the risk of central serous chorioretinopathy
Source: PLoS One. 2026 Mar 30;21(3):e0343704. doi: 10.1371/journal.pone.0343704 (PMC13035135; doi:10.1371/journal.pone.0343704)
Supplement: S1 Table — Longitudinal changes in central macular thickness (CMT) measured by optical coherence tomography following intravitreal steroid injections, stratified by treatment type and underlying retinal condition. Data are presented as mean ± standard deviation with sample sizes in parentheses. P-values represent statistical significance of changes from last injection to each follow-up timepoint using paired t-tests. Abbreviations: CMT = Central Macular Thickness; DME = Diabetic Macular Edema; RVO = Retinal Vein Occlusion; SD = Standard Deviation. (DOCX) [file pone.0343704.s002.docx]

**S1 Table. Changes in Central Macular Thickness Following Intravitreal Steroid Injections**

Longitudinal changes in central macular thickness (CMT) measured by optical coherence tomography following intravitreal steroid injections, stratified by treatment type and underlying retinal condition. Data are presented as mean ± standard deviation with sample sizes in parentheses. P-values represent statistical significance of changes from last injection to each follow-up timepoint using paired t-tests.

Abbreviations: CMT = Central Macular Thickness; DME = Diabetic Macular Edema; RVO = Retinal Vein Occlusion; SD = Standard Deviation

| **Sub‑group** | **Time point** | **Dexamethasone implant (mean ± SD, n)** | **p** | **Triamcinolone acetonide (mean ± SD, n)** | **p** |
| --- | --- | --- | --- | --- | --- |
| Total | First injection | 542.1 ± 145.2 (153) | 0.0005 | 471.7 ± 148.9 (110) | 0.0031 |
|  | Last injection | 492.8 ± 181.2 (151) | — | 439.7 ± 146.4 (110) | — |
|  | 1 month | 326.0 ± 120.0 (96) | < 0.0001 | 325.6 ± 101.2 (90) | < 0.0001 |
|  | 3 months | 355.9 ± 139.8 (114) | < 0.0001 | 359.8 ± 136.2 (92) | < 0.0001 |
|  | 6 months | 349.1 ± 122.9 (100) | < 0.0001 | 379.1 ± 137.4 (80) | < 0.0001 |
| RVO | First injection | 533.7 ± 147.4 (44) | 0.9447 | 436.6 ± 143.7 (25) | 0.6894 |
|  | Last injection | 539.6 ± 208.4 (43) | — | 465.9 ± 169.7 (26) | — |
|  | 1 month | 352.0 ± 179.7 (26) | 0.0003 | 356.8 ± 117.0 (23) | 0.0056 |
|  | 3 months | 369.0 ± 172.3 (33) | 0.0004 | 405.3 ± 166.7 (22) | 0.0869 |
|  | 6 months | 355.6 ± 140.4 (26) | 0.0019 | 474.5 ± 178.3 (17) | 0.2553 |
| Uveitis | First injection | 618.5 ± 128.8 (11) | 0.0248 | 588.1 ± 125.1 (16) | 0.0281 |
|  | Last injection | 493.5 ± 136.8 (11) | — | 529.0 ± 164.3 (16) | — |
|  | 1 month | 313.0 ± 95.7 (7) | 0.0136 | 296.4 ± 54.8 (11) | 0.007 |
|  | 3 months | 369.1 ± 158.2 (10) | 0.0006 | 375.9 ± 140.3 (14) | 0.0019 |
|  | 6 months | 294.1 ± 59.5 (7) | 0.0168 | 407.4 ± 156.2 (13) | 0.0186 |
| DME | First injection | 540.2 ± 146.3 (91) | 0.0015 | 449.0 ± 145.7 (61) | 0.0015 |
|  | Last injection | 479.3 ± 172.5 (90) | — | 395.4 ± 106.4 (60) | — |
|  | 1 month | 315.0 ± 89.8 (59) | < 0.0001 | 316.5 ± 99.9 (50) | < 0.0001 |
|  | 3 months | 352.4 ± 122.4 (67) | < 0.0001 | 325.9 ± 108.4 (50) | < 0.0001 |
|  | 6 months | 354.3 ± 121.7 (65) | < 0.0001 | 330.4 ± 76.0 (46) | 0.0002 |
