# Supplementary material for: Intravitreal injections of corticosteroid and the risk of central serous chorioretinopathy
Source: PLoS One. 2026 Mar 30;21(3):e0343704. doi: 10.1371/journal.pone.0343704 (PMC13035135; doi:10.1371/journal.pone.0343704)
Supplement: Attachment — Submitted filename: PLOS one revision_Final.docx [file pone.0343704.s004.docx]

December 24, 2025

Manuscript ID: PONE-D-25-57894

Title: Intravitreal injections of corticosteroid and the risk of central serous chorioretinopathy

Dear Editor and Reviewers,

We sincerely appreciate the thoughtful and constructive feedback from both reviewers. Your comments have significantly improved the quality and clarity of our manuscript. All comments from the reviewers were very thoughtful and have helped us to improve our manuscript. We have carefully checked our manuscript and made appropriate revisions based on the reviewers’ suggestions.

A point-by-point response to the editor’s comments is presented below.

**Response to Reviewer #1**

**Reviewer Comment 1:**

**"This is a clinically important study addressing a mechanistically intriguing and very rare event. Prior evidence is mostly isolated case reports; a 21-year, zero-event cohort is highly reassuring. It would help if the authors more explicitly compare their upper 95% CI with the expected background incidence (e.g., estimated expected CSC cases in this population), to better frame the clinical impact."**

Response: We thank the reviewer for this excellent suggestion. We have added a more explicit quantitative comparison in the Discussion section, while acknowledging the statistical limitations of zero-event observations.

**Lines 298-314:**

“Applying this rate to our cohort over the 6-month surveillance period (2,336 patients × 0.5 years = 1,168 person-years), approximately 0.5-0.6 CSC cases would be expected based on general population incidence alone. While the upper bound of our 95% confidence interval (0.13%, representing a maximum of approximately 3 cases among 2,336 patients) cannot definitively exclude a modest risk increase, several lines of evidence suggest intravitreal steroids do not carry clinically meaningful CSC risk. First, CSC predominantly affects individuals aged between 28 and 68 years, with an average age of 43.[18] Given our cohort’s older age distribution, applying an incidence rate based on the general population may overestimate the expected number of cases, since the incidence rate of CSC decreases after middle age.[19] Second, the observation of zero events despite adequate sample size provides reassurance that any potential risk is not of the magnitude associated with systemic corticosteroid use, which increases CSC risk(OR 4.05 in meta-analysis; adjusted HR 1.81 in a nationwide cohort).[20,21] Third, our findings are strengthened by the complete absence of CSC even among patients receiving multiple repeated injections, which would be expected to amplify any dose-dependent risk if present. Therefore, while we cannot exclude all possibility of a small risk increase based on the confidence interval alone, we can suggest intravitreal steroid injections do not pose clinically significant CSC risk comparable to systemic corticosteroid administration."

20. Gäckle HC, Lang GE, Freissler KA, Lang GK. [Central serous chorioretinopathy. Clinical, fluorescein angiography and demographic aspects]. Ophthalmol Z Dtsch Ophthalmol Ges. 1998;95: 529–533. doi:10.1007/s003470050310

21.	Tsai D-C, Chen S-J, Huang C-C, Chou P, Chung C-M, Huang P-H, et al. Epidemiology of Idiopathic Central Serous Chorioretinopathy in Taiwan, 2001–2006: A Population-based Study. PLOS ONE. 2013;8: e66858. doi:10.1371/journal.pone.0066858

22.	Ge G, Zhang Y, Zhang Y, Xu Z, Zhang M. Corticosteroids usage and central serous chorioretinopathy: a meta-analysis. Graefes Arch Clin Exp Ophthalmol. 2020;258: 71–77. doi:10.1007/s00417-019-04486-w

23.	Rim TH, Kim HS, Kwak J, Lee JS, Kim DW, Kim SS. Association of Corticosteroid Use With Incidence of Central Serous Chorioretinopathy in South Korea. JAMA Ophthalmol. 2018;136: 1164–1169. doi:10.1001/jamaophthalmol.2018.3293

**Reviewer Comment 2:**

**"OCT availability and choroidal thickness methodology. For the CDW cohort, please clarify whether OCT data were directly used or whether CSC screening relied only on codes and free text. For the detailed cohort, specify: Whether EDI-OCT was used for all included eyes. What proportion of all intravitreal steroid–treated eyes in 2023–2024 had analyzable OCT and why others were excluded. This is important for understanding selection bias and how broadly imaging findings apply."**

Response:

We appreciate this important clarification request. We have revised the Methods section to address these concerns comprehensively.

Lines 103-106:

"Only the three suspected cases meeting both injection and diagnosis codes or free-text clinical notes underwent comprehensive manual review by qualified ophthalmologists (JRS, SJW) to determine the temporal and causal relationship between steroid injection and CSC development.

Regarding EDI-OCT usage, this has already been specified in the Methods section (Lines 135-136): "Enhanced depth imaging (EDI) mode was routinely employed to optimise choroidal visualisation."

We have now added information about the proportion of analyzable OCT data:

Lines 127-131

" Between January 2023 and October 2024, a total of 361 eyes received intravitreal steroid injections at our institution. Among these patients without concurrent or previous systemic corticosteroid use, 269 eyes were included in the final analysis after excluding 76 eyes with insufficient follow-up and 16 eyes with inadequate choroidal visualization on pre-injection OCT. These 16 eyes showed no CSC features on clinical assessment.”

**Reviewer Comment 3:**

**"Definition and adjudication of CSC. Please provide a clear operational definition of CSC (clinical + OCT ± angiography criteria) and describe the adjudication process (number of reviewers, how discrepancies were handled). This will reassure readers that..."**

Response:

We agree that a clear operational definition and adjudication process is essential. We have added the following to the Methods section:

Lines 106-110:

"CSC was defined as subretinal fluid on optical coherence tomography in the macular region without alternative explanation. When available, fluorescein angiography findings such as focal leakage points provided additional diagnostic confirmation. Moreover, temporal relationships with steroid injections were evaluated. Discrepancies were resolved through consensus discussion.”

**Reviewer Comment 4:**

**"Systemic/other steroid exposure and prior CSC. Since the main concern is "steroid-induced" CSC, more detail on other steroid exposures would strengthen the..."**

Response:

We thank the reviewer for highlighting this important methodological detail. We have clarified our exclusion criteria to the Methods section:

Lines 127-131

" Between January 2023 and October 2024, a total of 361 eyes received intravitreal steroid injections at our institution. Among these patients without concurrent or previous systemic corticosteroid use, 269 eyes were included in the final analysis after excluding 76 eyes with insufficient follow-up and 16 eyes with inadequate choroidal visualization on pre-injection OCT. These 16 eyes showed no CSC features on clinical assessment.”

**Response to Reviewer #2**

**Reviewer Comment 1:**

**"The abstract could benefit from being re-organized."**

Response:

We have comprehensively reorganized the abstract to improve flow and clarity. The revised abstract now follows a more logical structure:

Line 21-37:

“Central serous chorioretinopathy (CSC) is strongly associated with systemic corticosteroid use, but the risk following intravitreal steroid injections remains unclear, with limited evidence from isolated case reports. This study evaluated the association between intravitreal steroid injections and CSC development using a dual-approach design with Clinical Data Warehouse (CDW) analysis and choroidal thickness validation. We performed CDW analysis of 2,336 patients receiving intravitreal triamcinolone acetonide or dexamethasone implant over 21 years (April 2003–October 2024), screening for CSC within 6 months post-injection. Additionally, we conducted detailed subfoveal choroidal thickness analysis in 269 eyes (January 2023–October 2024) at baseline, 1, 3, and 6 months post-injection using optical coherence tomography. No confirmed CSC cases occurred in either cohort (0/2,336 patients, 95% CI: 0–0.13%; 0/269 eyes). Contrary to the choroidal thickening characteristic of CSC, both steroid formulations produced significant choroidal thinning at 1 month: dexamethasone implant (227.0±65.4μm to 217.9±62.0μm, p<0.0001) and triamcinolone acetonide (219.8±78.7μm to 218.6±77.7μm, p<0.0001). Only 4.9% of eyes showed ≥10% choroidal thickening, and none developed CSC features. Both formulations achieved significant macular edema reduction through 6 months. Intravitreal corticosteroids showed no confirmed CSC cases over two decades with choroidal thinning rather than the thickening characteristic of CSC, suggesting distinct pathophysiological mechanisms and a favorable safety profile regarding CSC development.”

**Reviewer Comment 2:**

**"What was the rationale for limiting the search to 6 months after intravitreal injection?"**

Response:

We thank the reviewer for this important question. We have added justification for the 6-month timeframe based on existing case report literature. The following has been added to the Methods section:

Lines 97-99:

"6 months post-injection based on temporal patterns reported in previous case reports[11–14] and pharmacokinetic studies of dexamethasone implants showing sustained drug release for up to 6 months with negligible residual intraocular steroid thereafter.[16,17]”

11.	Imasawa M, Ohshiro T, Gotoh T, Imai M, Iijima H. Central serous chorioretinopathy following vitrectomy with intravitreal triamcinolone acetonide for diabetic macular oedema. Acta Ophthalmol Scand. 2005;83: 132–133. doi:10.1111/j.1600-0420.2005.00379.x

12.	Baumal CR. Central Serous Chorioretinopathy Associated With Periocular CorticosteroidInjection Treatment for HLA-B27–Associated Iritis. Arch Ophthalmol. 2004;122: 926. doi:10.1001/archopht.122.6.926

13.	Georgalas I, Petrou P, Pagoulatos D, Papaconstantinou D, Tservakis I. Central serous chorioretinopathy in the fellow eye as a complication of intravitreal dexamethasone implant for the treatment of Irvine‐Gass syndrome. Clin Exp Optom. 2016;99: 601–603. doi:10.1111/cxo.12420

14.	Noh GM, Nam KY, Lee SU, Lee SJ. Central Serous Chorioretinopathy Following Intravitreal Dexamethasone Implant. Korean J Ophthalmol. 2019;33: 392. doi:10.3341/kjo.2018.0115

16.	Chang-Lin J-E, Burke JA, Peng Q, Lin T, Orilla WC, Ghosn CR, et al. Pharmacokinetics of a Sustained-Release Dexamethasone Intravitreal Implant in Vitrectomized and Nonvitrectomized Eyes. Invest Ophthalmol Vis Sci. 2011;52: 4605–4609. doi:10.1167/iovs.10-6387

17.	Chang-Lin J-E, Attar M, Acheampong AA, Robinson MR, Whitcup SM, Kuppermann BD, et al. Pharmacokinetics and pharmacodynamics of a sustained-release dexamethasone intravitreal implant. Invest Ophthalmol Vis Sci. 2011;52: 80–86. doi:10.1167/iovs.10-5285

**Reviewer Comment 3:**

**"Clarify in the methods whether patients received both 'triamcinolone acetonide and dexamethasone implant' (as stated at the moment) or one of the two."**

Response:

We apologize for this ambiguity. We have clarified that patients received one of the two steroid formulations, not both concurrently. The following revisions have been made:

Lines 122-124:

"A detailed retrospective analysis was conducted on patients who received intravitreal steroid injections with either triamcinolone acetonide 40 mg (Maqaid, Hanmi Pharm., Seoul, Korea) or dexamethasone implant 700 mcg (Ozurdex, Korea AbbVie Ltd., Seoul, Korea)) between January 2023 and October 2024. "

**Reviewer Comment 4:**

**"Loss to follow-up: is all post-injection follow-up performed in-house? How many patients are missing data post-injection?"**

**Response:** We have now added information about the proportion of analyzed OCT data:

Lines 127-131:

" Between January 2023 and October 2024, a total of 361 eyes received intravitreal steroid injections at our institution. Among these patients without concurrent or previous systemic corticosteroid use, 269 eyes were included in the final analysis after excluding 76 eyes with insufficient follow-up and 16 eyes with inadequate choroidal visualization on pre-injection OCT. These 16 eyes showed no CSC features on clinical assessment."

**Reviewer Comment 5:**

**"The use of SFCT as a surrogate for CSC is reasonable, however - CSC often presents in a non-subfoveal location, which should be acknowledged."**

Response:

This is an excellent point that we have added to the limitations

Line 383-385:

“Furthermore, while we used subfoveal choroidal thickness as a surrogate marker for CSC development, this approach may not capture cases where CSC presents with non-subfoveal subretinal fluid and predominantly extrafoveal choroidal thickening."

**Reviewer Comment 6:**

**"There is no control group of similar patients receiving no or different treatment - could the observed thinning reflect the natural history of the underlying pathology rather than steroid effect?"**

Response:

We appreciate this thoughtful consideration of alternative explanations. We have enhanced our Discussion to address this important point:

Lines 329-336:

“While our study lacks a concurrent control group, making it impossible to definitively distinguish steroid effects from natural disease progression, the key finding for our research question is not whether choroidal thinning represents steroid effect versus natural history(which typically shows stable choroidal thickness when untreated)[26], but rather that choroidal thickening (the hallmark feature of CSC) was not observed following intravitreal steroid injection. The absence of choroidal thickening and CSC development across all disease subgroups provides supporting evidence against a causal relationship between intravitreal steroid administration and CSC, which was the primary objective of our investigation.”

27.	Yiu G, Manjunath V, Chiu SJ, Farsiu S, Mahmoud TH. Effect of Anti–Vascular Endothelial Growth Factor Therapy on Choroidal Thickness in Diabetic Macular Edema. Am J Ophthalmol. 2014;158: 745-751.e2. doi:10.1016/j.ajo.2014.06.006

**Reviewer Comment 7:**

**"In Table 2, parentheses around the n values are misplaced."**

Response:

We have carefully reviewed Table 2 and corrected the parentheses placement.

**Reviewer Comment 8:**

**"Please report the range of ages (not just mean ± SD) to clarify whether patients in the typical CSC age group were included, and if not - address."**

Response: Thanks for your comments. We added the range of ages at Table 1.

Thank you for your detailed feedback and consideration of our submitted paper. Your feedback has helped us address important issues and significantly improve our manuscript. We believe these comprehensive revisions have enhanced the scientific rigor, clarity, and clinical relevance of our work. We hope our responses have adequately addressed your comments and that our revised manuscript is now suitable for publication in PLOS ONE.

Sincerely,

Se Joon Woo, MD, PhD

Department of Ophthalmology

Seoul National University Bundang Hospital

82, Gumi-ro 173beon-gil, Bundang-gu, Seongnam, Gyeonggi-do, South Korea, 13620

Tel: +82-31-787-7377

Email: sejoon1@snu.ac.kr
