# Supplementary material for: Intravitreal injections of corticosteroid and the risk of central serous chorioretinopathy
Source: PLoS One. 2026 Mar 30;21(3):e0343704. doi: 10.1371/journal.pone.0343704 (PMC13035135; doi:10.1371/journal.pone.0343704)
Supplement: Attachment — Submitted filename: PLOS one minor revision_2601.docx [file pone.0343704.s005.docx]

January 21, 2026

Manuscript ID: PONE-D-25-57894

Title: Intravitreal injections of corticosteroid and the risk of central serous chorioretinopathy

Dear Editor and Reviewers,

We sincerely thank the reviewer for the continued careful evaluation of our manuscript and for the constructive suggestions that further improve its transparency and clinical relevance. We have addressed all comments in detail as outlined below.

**Response to Reviewer #1**

**Reviewer Comment 1: I would like to thank the authors for addressing an important yet clinically vague topic and for carefully revising the manuscript**

**"Free-text screening methodology in the CDW cohort**

**Given that OCT images were not systematically reviewed for the majority of the CDW cohort, the accuracy of CSC detection relies heavily on diagnosis codes and free-text searches. To improve transparency and reproducibility, the Methods section would benefit from a more detailed description of the free-text search strategy. Specifically, listing the exact search terms (and whether variants, abbreviations, or non-English terms were included), similar to how search strings are reported in systematic reviews, would strengthen confidence in the completeness of case ascertainment."**

Response: We appreciate this valuable suggestion to enhance the transparency and reproducibility of our search methodology. We have revised the Methods section to provide comprehensive details of our free-text search strategy, including the exact search terms and inclusion of abbreviations and Korean equivalents.

**Line 94-98**

These patients were then screened for CSC diagnosis following steroid injection using both structured diagnosis codes containing 'Central serous chorioretinopathy' and unstructured free-text clinical notes containing CSC-related terms ('CSC', ‘CSCR’, 'central serous chorioretinopathy' and Korean equivalents) within 6 months post-injection based on temporal patterns reported in previous case reports

**“The relationship between Intra virtual corticosteroid exposure and central serous chorioretinopathy remains a source of uncertainty in daily practice. Many clinicians continue to avoid intravitreal steroids in patients with pachychoroid spectrum disease on their differential, despite the evidence being largely limited to isolated case reports.**

**The Discussion could be further strengthened by explicitly framing how these findings together with the fact that CSCR wasn't raised as a safety issue in the pivotal trials approving these preparations/implant should influence clinical decision-making. Otherwise, suggest which additional work is required to make such a conclusion”**

Response: We thank the reviewer for this insightful comment regarding the clinical implications of our findings. We have added a new paragraph to the Discussion section that addresses this concern.

**Line 361-371:**

“Moreover, our results align with the safety profiles reported in pivotal clinical trials for intravitreal corticosteroids. The FAME studies, GENEVA studies, and DRCR.net trial did not report CSC as a safety concern.[32–36] This concordance between our real-world evidence and controlled trial data, combined with the mechanistic finding of choroidal thinning rather than thickening, suggests that the association reported in isolated case reports may represent idiosyncratic reactions in genetically predisposed individuals rather than a class effect. From a clinical decision-making perspective, routine avoidance of intravitreal corticosteroids based solely on theoretical CSC concerns may not be warranted in the general patient population. However, heightened monitoring remains prudent in patients with pre-existing pachychoroid features, documented CSC history, or other known risk factors such as systemic corticosteroid use. Future prospective studies are needed to examine CSC development in these high-risk subgroups.”

32.	Campochiaro PA, Brown DM, Pearson A, Chen S, Boyer D, Ruiz-Moreno J, et al. Sustained delivery fluocinolone acetonide vitreous inserts provide benefit for at least 3 years in patients with diabetic macular edema. Ophthalmology. 2012;119: 2125–2132. doi:10.1016/j.ophtha.2012.04.030

33.	Campochiaro PA, Brown DM, Pearson A, Ciulla T, Boyer D, Holz FG, et al. Long-term benefit of sustained-delivery fluocinolone acetonide vitreous inserts for diabetic macular edema. Ophthalmology. 2011;118: 626-635.e2. doi:10.1016/j.ophtha.2010.12.028

34.	Haller JA, Bandello F, Belfort R, Blumenkranz MS, Gillies M, Heier J, et al. Randomized, sham-controlled trial of dexamethasone intravitreal implant in patients with macular edema due to retinal vein occlusion. Ophthalmology. 2010;117: 1134-1146.e3. doi:10.1016/j.ophtha.2010.03.032

35.	Haller JA, Bandello F, Belfort R, Blumenkranz MS, Gillies M, Heier J, et al. Dexamethasone intravitreal implant in patients with macular edema related to branch or central retinal vein occlusion twelve-month study results. Ophthalmology. 2011;118: 2453–2460. doi:10.1016/j.ophtha.2011.05.014

36.	Diabetic Retinopathy Clinical Research Network. A randomized trial comparing intravitreal triamcinolone acetonide and focal/grid photocoagulation for diabetic macular edema. Ophthalmology. 2008;115: 1447–1449, 1449.e1–10. doi:10.1016/j.ophtha.2008.06.015

**Response to Reviewer #2**

**“The authors have addressed all comments satisfactorily. The revisions clarify methodology, justify the 6-month follow-up window, transparently report exclusions and follow-up, and appropriately acknowledge key limitations, including the use of SFCT and the lack of a control group. Overall, the changes improve clarity and interpretability, and the manuscript is suitable for publication.”**

Response:

We thank Reviewer #2 for their positive evaluation and for confirming that all previous comments have been satisfactorily addressed.

Thank you for your detailed feedback and consideration of our submitted paper. Your feedback has helped us address important issues and significantly improve our manuscript. We believe these comprehensive revisions have enhanced the scientific rigor, clarity, and clinical relevance of our work. We hope our responses have adequately addressed your comments and that our revised manuscript is now suitable for publication in PLOS ONE.

Sincerely,

Se Joon Woo, MD, PhD

Department of Ophthalmology

Seoul National University Bundang Hospital

82, Gumi-ro 173beon-gil, Bundang-gu, Seongnam, Gyeonggi-do, South Korea, 13620

Tel: +82-31-787-7377

Email: sejoon1@snu.ac.kr
